# On‐line targeted metabolomics for real‐time monitoring of relevant compounds in fermentation processes

**DOI:** 10.1002/bit.28599

**Published:** 2023-11-22

**Authors:** Joan Cortada‐Garcia, Jennifer Haggarty, Stefan Weidt, Rónán Daly, S. Alison Arnold, Karl Burgess

**Affiliations:** ^1^ School of Biological Sciences, Institute of Quantitative Biology, Biochemistry and Biotechnology University of Edinburgh Edinburgh UK; ^2^ Glasgow Polyomics University of Glasgow Glasgow UK; ^3^ Ingenza Ltd. Roslin Innovation Centre Roslin UK

**Keywords:** bioprocess monitoring, fermentation monitoring, on‐line metabolomics, process analytical technologies, real‐time metabolomics

## Abstract

Fermentation monitoring is a powerful tool for bioprocess development and optimization. On‐line metabolomics is a technology that is starting to gain attention as a bioprocess monitoring tool, allowing the direct measurement of many compounds in the fermentation broth at a very high time resolution. In this work, targeted on‐line metabolomics was used to monitor 40 metabolites of interest during three *Escherichia coli* succinate production fermentation experiments every 5 min with a triple quadrupole mass spectrometer. This allowed capturing high‐time resolution biological data that can provide critical information for process optimization. For nine of these metabolites, simple univariate regression models were used to model compound concentration from their on‐line mass spectrometry peak area. These on‐line metabolomics univariate models performed comparably to vibrational spectroscopy multivariate partial least squares regressions models reported in the literature, which typically are much more complex and time consuming to build. In conclusion, this work shows how on‐line metabolomics can be used to directly monitor many bioprocess compounds of interest and obtain rich biological and bioprocess data.

## Graphical abstract

In this work, targeted on‐line metabolomics was used to directly monitor 40 process‐relevant compounds during three *Escherichia coli* succinate production fermentation experiments every 5 min with a triple quadrupole mass spectrometer. Cortada‐Garcia and co‐workers describe how this novel process analytical technologies tool allows to easily obtain rich biological and bioprocess data with little post‐acquisition data treatment.

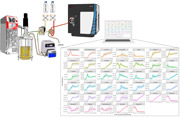

## 1 INTRODUCTION

### 1.1 Fermentation monitoring

Bioprocess monitoring is a tool used at research and manufacturing scale to better characterize industrial fermentation and cell culture processes and enables the control of different parameters within desired values. Temperature, pH, and dissolved oxygen (DO) are typical parameters monitored in bioreactor processes, and are often maintained at predefined set values to modulate cellular and product formation kinetics, metabolic regulation, product solubility, and so forth. (Mandenius & Titchener‐Hooker, 2013; Vogel & Todaro, 2014). The oxygen and carbon dioxide entering and leaving the gas phase of the bioreactor are also often monitored and provide key information of the metabolic activity of the cultured cells and can indirectly reveal crucial process information such as substrate starvation (Pepeliaev & Ehebauer, 2022; Stratton et al., 1998).

Biological systems, however, are very complex, involving a plethora of biochemical species in intertwined metabolic pathways. For this reason, despite the usefulness of measuring the off‐gas O_2_ and CO_2_, the opportunity to analyze additional chemical species in the fermentation broth is a target for intensive research in the field of process analytical technologies (PAT) for bioprocess development and optimization. High‐performance liquid chromatography (HPLC) and vibrational spectroscopy are–respectively–commonly used technologies for the off‐line and on‐/in‐line monitoring of key process metabolites in the bioreactor broth. On‐ and in‐line tools are particularly sought after for process monitoring as they provide highly time‐resolved analysis, allowing better process characterization and faster response times upon biological/process changes. Although vibrational spectroscopy technologies such as Raman and infrared (IR) have been successfully used for real‐time monitoring purposes in bioreactors, these are generally limited to analyzing a small number of target compounds, that is, usually no more than six metabolites are reported in literature studies (Abu‐Absi et al., 2011; Arnold et al., 2003; Nascimento et al., 2017; Iversen et al., 2014; Rodrigues et al., 2018; Schenk et al., 2008; Zu et al., 2017).

Furthermore, the use of vibrational spectroscopy for bioprocess monitoring is based on the deconvolution of overlapping spectral peaks that are not associated with individual molecular species but rather with functional chemical groups. Therefore, this requires the development of laborious chemometric models such as partial least squares (PLS) regression to distinguish the different compounds in the mixture. Finally, these models have the risk of heavily depending on the exact conditions that they are developed with, such as the organism, medium composition, pH or stirring speed used in the process with model transferability being a known challenge in industry and reported in the literature (Esmonde‐White et al., 2017; Roggo et al., 2007; Roychoudhury et al., 2007). This significantly limits the applicability of models developed in research laboratories and their transferability to pilot and manufacturing scale, where generating data for model calibration can be prohibitively expensive.

Compared to these techniques, mass spectrometry (MS) offers a much wider detection capacity, making it an attractive alternative PAT tool for bioprocess monitoring. Several publications have shown the use of MS for fermentation headspace analysis, allowing the monitoring of many compounds (Berbegal et al., 2020; Custer et al., 2003; Luchner et al., 2012; Tejero Rioseras et al., 2017). However, this approach is limited to volatile molecules, whereas fermentation monitoring with MS directly from the liquid broth is a much less developed approach. The authors recently demonstrated the use of untargeted on‐line metabolomics (RTMet) directly sampling from the liquid phase as a bioprocess monitoring tool with an *Escherichia coli* fermentation process of succinate production (Cortada‐Garcia et al., 2022). In the study, the authors used a high‐resolution Orbitrap mass spectrometer, which allows the detection of metabolites with a mass tolerance of 5 ppm. Due to these high‐resolution accurate mass characteristics, Orbitrap MS can simultaneously detect a vast array of metabolites and distinguish very similar masses, enabling the identification of key process metabolites and biomarkers, which makes the system well‐suited for untargeted analysis. Once these compounds of interest have been identified, a targeted metabolomics method can be developed to specifically monitor them in a more quantitative manner.

Targeted metabolomics is the analysis of a predefined specific set of compounds using metabolomics. Triple quadrupole (QQQ) mass spectrometers are the most popular option for performing targeted metabolomics analyzes, mainly due to their wide linear dynamic range and speed in transitioning between the different target metabolites to analyze (Gross, 2017), which allow better metabolite quantification than other types of MS analyzers.

In this study, targeted RTMet was used to monitor 40 process‐relevant compounds during an *E. coli* succinate fermentation process using a QQQ mass spectrometer. As MS can distinctly differentiate between different metabolites, simple univariate models can be used to monitor the concentration of compounds of interest. These are much simpler than multivariate PLS models that rely on vibrational spectroscopy. Altogether, this study shows how on‐line metabolomics can directly monitor a large number of process‐relevant metabolites in a bioreactor and how high‐resolution time‐course biological and process data can be derived from this technology with little post‐acquisition data treatment.

## 2 MATERIALS AND METHODS

**Figure 1:**
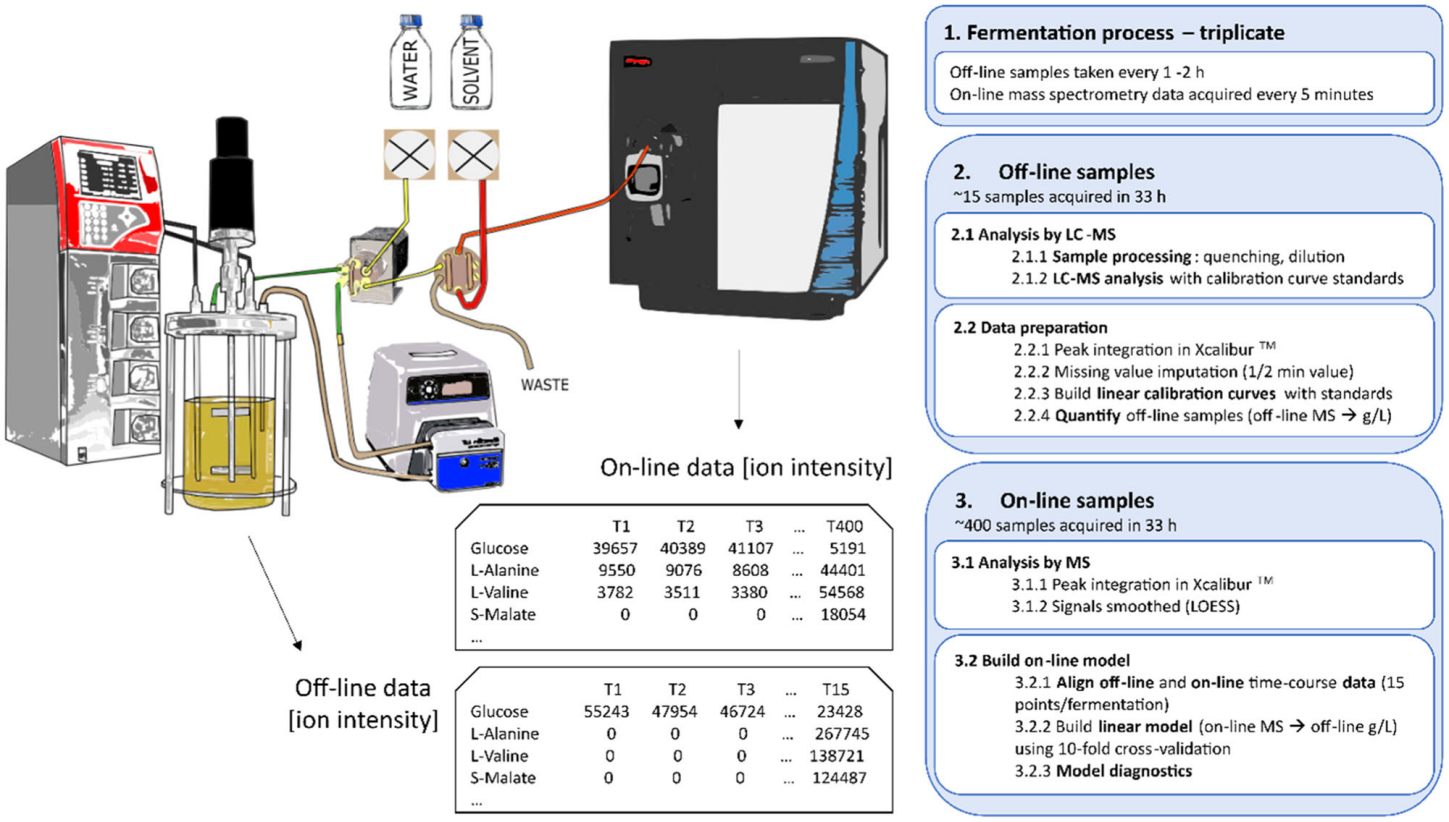
Process to build the linear regression models to monitor the concentration of targeted metabolites on‐line.

A schematic of the experimental setup used in this work to develop the regression models for monitoring the concentration of metabolites on‐line is shown in Figure 1. The specific parts of the schematic diagram are explained in more detail in the corresponding subsections below.

### 2.1 Bacterial strain and growth media

All experiments described in this article were carried out using the proprietary industrial *E. coli* strain (Ingenza Ltd.) and growth media described by Cortada‐Garcia et al. (2022) and in the Supporting Information.

### 2.2 Fermentation process conditions

All fermentation experiments were carried out in a 5 L Applikon stirred tank fermenter controlled with a FerMac 320 (Electrolab Biotech Ltd.) coupled to a FerMac 368 gas analyzer for off‐gas analysis. The fermentation consisted of a dual‐phase succinate production process as described by Cortada‐Garcia et al. (2022). A biomass filtration probe (TRACE Analytics GmbH) equipped with a polypropylene membrane (0.2 µm pore size) was installed in the fermenter through the headplate and submerged in the fermentation broth. Cell‐free fermentation broth was continuously withdrawn from the probe and recirculated into the fermenter, while a small 50 μL fraction of the broth was automatically sent to a mass spectrometer every 5 min for RTMet analysis (see Section 2.4).

#### 2.2.1 Inoculum

Fermentation seed cultures were prepared by inoculating 25 µL of *E. coli* cell bank into 100 mL of growth medium in a 500 mL baffled shake flask and incubated at 37℃ and 215 rpm for 18.15 ± 0.5 h.

#### 2.2.2 Aerobic batch phase for biomass growth

The fermentation was started by inoculating 100 mL of overnight seed culture into 3 L of growth medium in the 5 L fermenter for a starting OD_600_ of 0.20 ± 0.04. During biomass growth the conditions were maintained at 37℃ temperature, 600–1200 rpm agitation (controlled to keep the DO > 30%), 4.00 L/min air (1.33 vvm) and pH 7.0 ± 0.1, controlled with 2.00 M H_2_SO_4_ and 28% w/v NH_4_OH.

#### 2.2.3 Anaerobic succinate production phase

At the beginning of the production phase, glucose from a 500 g/L solution and sodium bicarbonate from a 100 g/L solution were added to the fermenter as a single bolus addition to a final concentration of 20 and 5 g/L respectively in the vessel, as described by (Wu et al., 2007). The sodium bicarbonate provides soluble CO_2_, which is required for the conversion of PEP to oxaloacetate (see Figure S1 in the Supporting Information) (Thakker et al., 2012). Once the glucose and sodium bicarbonate were added to the fermenter, the sparged air was replaced by pure (99.8%) CO_2_ at 0.50 L/min (0.17 vvm), agitation was set to 600 rpm to prevent fouling of the biomass filtration probe, temperature at 37℃ and pH at 7.0 ± 0.1 controlled with 2.00 M H_2_SO_4_ and 28% w/v NH_4_OH.

### 2.3 Biomass measurement

Biomass levels were reported as OD_600_ and wet cell weight (WCW). The former was the measured optical density at 600 nm wavelength. The latter was determined by spinning down 1 mL of sample for 5 min at 14,462 × *g* twice in a preweighed Eppendorf tube, removing the supernatant and weighing the resulting pellet. The weight of the pellet in g/L was calculated from gravimetric difference.

### 2.4 On‐line metabolomics

On‐line metabolomics was conducted by connecting the fermenter to a TSQ Quantiva triple quadrupole (Thermo Scientific) mass spectrometer with the fluidics system previously described by Cortada‐Garcia et al. (2022) but the fermentation broth samples were subtracted from the bioreactor using a biomass filtration probe (TRACE Analytics GmbH) equipped with a polypropylene membrane. The filtration efficiency of the probe was measured to be at least 99.9801% ± 0.0037% (see Table S2 in the Supporting Information), therefore, the samples analyzed should consist of mostly cell‐free medium (referred to “cell‐free medium” for simplicity). A Masterflex L/S (Cole‐Parmer) high‐performance pump was used to continuously withdraw cell‐free broth from the fermenter for automatic metabolomics analysis and it was operated at a low flow rate of 1.5 mL/min to prevent cells from entering the filtration probe–as indicated by the filtration probe manufacturer. Due to the flow rate of the pump, the total traveling time from the bioreactor to the mass spectrometer was 31.5 min. This time difference was taken into account when analyzing the data and comparing off‐line and on‐line data. Despite this time offset, as fermentation broth was continuously withdrawn from the bioreactor, samples were analyzed every 5 min. Furthermore, the significantly reduced concentration of cells in the sample should minimize the chances of biochemical modifications of the metabolites in the sample during this traveling time, particularly in organisms like *E. coli*, where the secretion of enzymes to the extracellular space is uncommon. The fact that the concentration of products of anaerobic metabolism (such as succinate, pyruvate, and fumarate) measured on‐line did not increase during the aerobic phase of the process further supports that the sample did not undergo significant biochemical conversion during the travel time from the bioreactor to the mass spectrometer.

#### 2.4.1 Mass spectrometer parameters

Gas‐phase ions were generated using an Ion Max NG source (Thermo Scientific) with a HESI II probe. The mass spectrometer was operated using a multiple reaction monitoring (MRM) method in polarity switching mode (see Supplementary file MRM table. xlsx) with a spray voltage of ±3.5 kV. HESI probe temperature was set to 25℃, ion transfer tube temperature 325℃, sheath gas flow rate 50 a.u., auxiliary gas 10 a.u. and sweep gas 1 a.u. Resolution for both Q1 and Q3 was set at a FWHM of 0.7 *m/z*, cycle time was 0.8 s and collision induced dissociation fragmentation in Q2 was done under 1.5 mTorr pressure.

### 2.5 Off‐line metabolomics

At specific time points throughout the fermentation process, 1 mL fermentation samples were taken for off‐line metabolomics analysis. The samples were collected into prechilled microtubes, immediately quenched with 3 µL of 5 M H_2_SO_4_ and subsequently spun down at 4℃ and 13,000 × *g* for 10 min. The supernatants were collected as extracellular fractions and stored at −80℃ until further liquid chromatography mass spectrometry (LC‐MS) analysis. During handling, the microtubes were kept on ice. Once all off‐line samples from the different fermentation replicates had been collected, these were prepared for MS analysis by diluting 10 µL of sample into 390 µL of 1:3:1 chloroform:methanol:water (C:M:W). The samples were then vortexed for 5 s, mixed in a rotary shaker for 5 min at 4℃ and then centrifuged for 3 min at 13,000 × g and 4℃. At this point, 360 µL of sample were transferred into a new microtube and stored at −80℃ until LC‐MS analysis. During handling, the 1:3:1 C:M:W extraction solvent and the samples were kept on ice.

#### 2.5.1 Off‐line LC‐MS analysis

Metabolite separation was performed with a Thermo Scientific^TM^ UltiMate^TM^ 3000 UHPLC system using a polymeric zwitterionic hydrophilic interaction liquid chromatography (ZIC®‐pHILIC) column (Merck SeQuant®) (150 mm × 4.6 mm, 5 µm particle size). A linear gradient was applied to the column, running from 80% to 20% solvent B over 15 min, followed by a 2 min wash with 5% solvent B, and 9 min re‐equilibration with 80% solvent B, where solvent B was acetonitrile and solvent A (the remaining percentage) was 20 mM ammonium carbonate in water (pH 9.16). The total flow rate was 300 µL/min, column temperature was maintained at 40℃, sample injection volume was 10 µL, samples were maintained at 4℃ for the duration of the analysis and a HESI II probe was used on the ion source.

Metabolite detection was done using the same MS parameters as described for on‐line metabolomics, but the HESI probe temperature was set to 350℃ and mass resolution was set at a FWHM of 0.7 and 1.2 *m/z* respectively for Q1 and Q3.

### 2.6 Metabolomics data processing and analysis

Both on‐line and off‐line metabolomics raw data was processed with the software Xcalibur (Version 4.2.28.14) using the ICIS peak detection method with the following parameters: one smoothing point, a baseline window of 40, area noise factor of five, a peak noise factor of 10 and minimum peak height threshold of three signal‐to‐noise ratio. After processing, metabolite features were extracted as a. csv file. On‐line data was smoothed in the statistical software environment R (Version 3.6.1) using the ggplot2 package (Version 3.3.3; (Wickham, 2016)) with a locally estimated scatterplot smoothing (LOESS) method with a span between 0.15 and 0.50, depending on the metabolite. Missing value imputation was performed as described in the Supporting Information.

### 2.7 Estimating the absolute concentration of off‐line samples

Mixes containing different metabolites of interest for the bioprocess were prepared at known concentrations as reference standards. A series of dilutions of these standards was prepared in LC‐MS‐grade water and each dilution was analyzed by LC‐MS in duplicate, generating linear calibration curves for each metabolite in the mixes, correlating MS peak area with metabolite concentration. The relationship between these two variables was modeled by linear regression using the least squares method in Microsoft® Excel® (Version 2201) using the LINEST function and the calibration curves were used to calculate the concentration of metabolites in the off‐line fermentation samples. Negative concentration values (as calculated from the calibration curves) were imputed as zeros. To avoid batch‐to‐batch variation, the series of dilutions of reference standards and the off‐line fermentation samples were analyzed on the same LC‐MS run.

### 2.8 Modeling metabolite concentration from on‐line MS data

Real‐time metabolite concentration was modeled using on‐line metabolomics data. A schematic of the process followed to build the model is described in Figure 1. In short, three replicate fermentation experiments were performed. Off‐line extracellular samples were taken every 1–2 h and on‐line measurements were automatically acquired every 5 min. Off‐line samples were processed and analyzed as described in Section 2.5 and absolute metabolite concentrations were estimated using calibration curves built using reference standards as detailed in Section 2.7.

To estimate the concentration of metabolites from the on‐line metabolomics data univariate linear models were built for each metabolite using the metabolite concentration of the off‐line samples as the independent variable (*x*) and on‐line MS peak area as the dependent variable (*y*). The on‐line MS data was previously smoothed as detailed in Section 2.6 to reduce the effect of outliers. As the number of data points in the on‐line data set is about 27 times larger than the off‐line one (approximately 400–15), both data sets were aligned by the time of fermentation and only the 15 data points of the on‐line metabolomics data set that corresponded to the 15 off‐line samples were used to build the univariate linear regression models (43 data points in total with the three replicates). Ten‐fold cross‐validation was used to train and test the linear models using the caret package (Kuhn et al., 2021) in the statistical software environment R (Version 4.2.2) using the "trainControl" function. Model performance was assessed with the coefficient of determination (*R* ^2^), root‐mean‐square error (RMSE) and root‐mean‐square relative error (RMSRE) as follows:

$$R^{2}=1-\frac{\sum_{i=1}^{n}{\left(y_{i}-\hat{y_{i}}\right)}^{2}}{\sum_{i=1}^{n}{\left(y_{i}-\bar{y}\right)}^{2}},$$

$$\mathrm{RMSE}=\sqrt{\frac{1}{n}\sum_{i=1}^{n}{\left(c_{i}-\hat{c_{i}}\right)}^{2}},$$

$$\mathrm{RMSRE}=\frac{\mathrm{RMSE}}{\bar{c}}100,$$

where $y_{i}$ and $\hat{y_{i}}$ are the measured and model‐predicted MS data of the *i*‐th sample; $\overset{®}{y}$ is the mean MS value across n samples; $c_{i}$ and $\hat{c_{i}}$ are the measured and model‐predicted off‐line concentrations of the *i*‐th sample and $\overset{®}{c}$ is the mean off‐line concentration across n samples.

## 3 RESULTS AND DISCUSSION

### 3.1 Triplicate succinate fermentations

**Figure 2:**
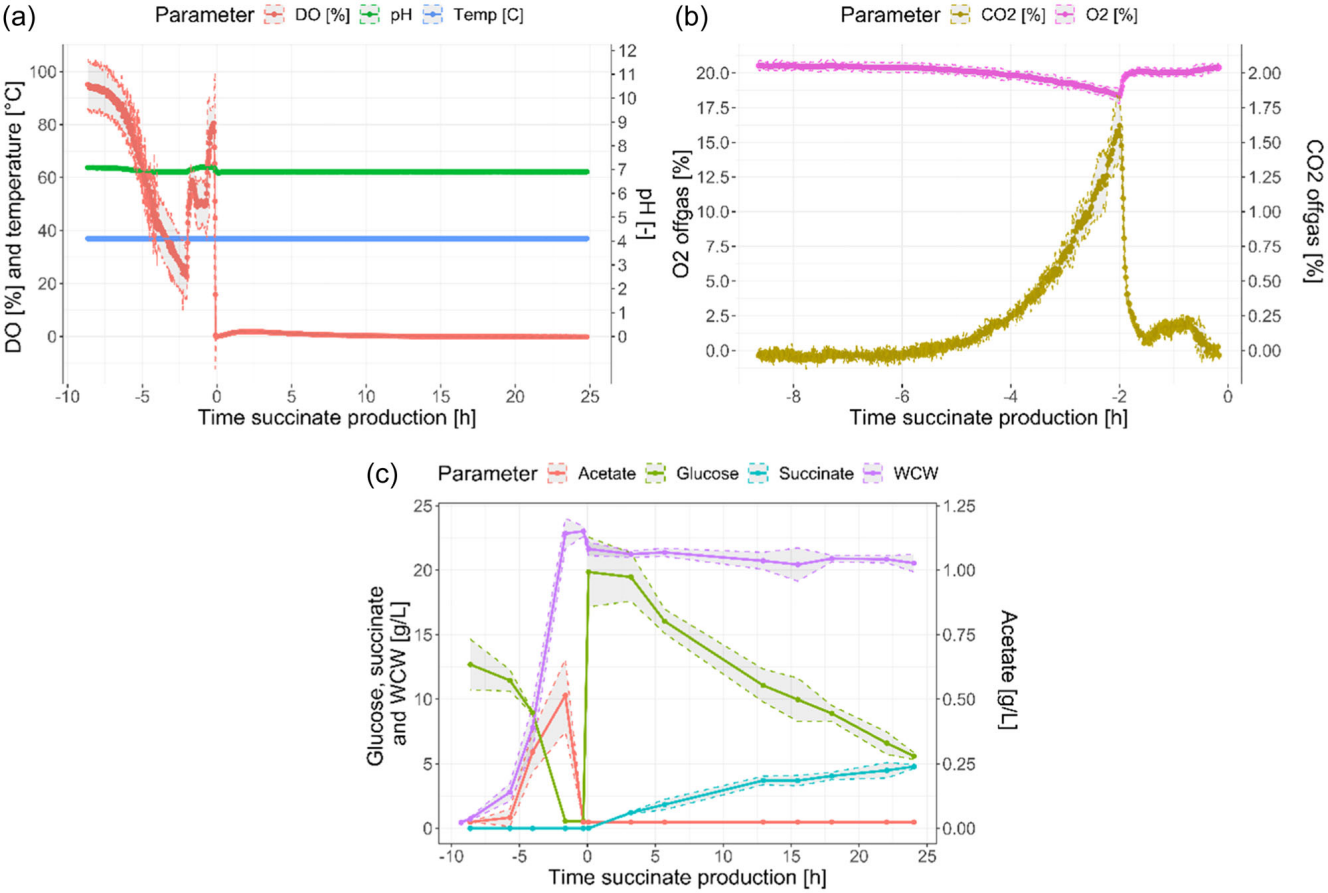
Dissolved oxygen (DO), pH, and temperature (a) of the three fermentation replicates that were monitored with targeted RTMet. The off‐gas CO_2_ and O_2_ (b) is only shown during the batch growth phase because during the production phase it was constant at 100% CO_2_. Acetate, glucose, succinate and the biomass wet cell weight (WCW) are also shown to help understand the different bioprocess phases (c). The 95% confidence intervals are shown with a gray area. Time is indicated with respect to the beginning of the succinate production phase for a better comparison of the three replicates.

An *E. coli* succinate fermentation process was run in triplicate. The process consisted of an initial aerobic batch phase for biomass formation followed by an anaerobic succinate production phase. Figure 2 shows the DO, pH, temperature, off‐gas CO_2_ and O_2_, and some of the main process compounds of the three replicates monitored during the process. During the batch phase, the biomass concentration increases exponentially, leading to an exponential decrease in DO in the fermentation broth. At the same time, the O_2_ and CO_2_ in the gas phase respectively decrease and increase exponentially. During the last 5 h of the batch phase, the DO decrease slows down due to the process control implemented, which increases the impeller stirring speed up to 1200 rpm (not shown) to maintain the DO setpoint (DO ≥ 30%), thus increasing the oxygen mass transfer from the gas to the liquid phase.

Two hours before the beginning of the succinate production phase the glucose is depleted, stopping aerobic metabolic activity. Consequently, biomass growth, O_2_ consumption and CO_2_ formation stop, characterized by a stable biomass concentration, a spike in DO and off‐gas O_2_, and a sharp drop in off‐gas CO_2_. At this point (1.5–2.0 h before the end of the batch phase), metabolic activity is briefly restored, typically caused by the consumption of compounds of energy storage, such as acetate.

During the anaerobic succinate production phase, additional glucose (20 g/L) and sodium bicarbonate (5 g/L) are added to the fermentation medium, and air sparging is replaced by CO_2_ sparging. Under these anaerobic conditions, glucose is converted into organic acids, including succinate, the main process product. During this phase, the DO is maintained at 0% and the off‐gas O_2_ and CO_2_ at 0% and 100%, respectively. The latter are not shown because the off‐gas analyzer was not calibrated for such high CO_2_ concentrations. The pH and temperature were kept constant at 7.0 and 37℃ throughout the whole process.

Throughout the fermentation process, relevant process metabolites were monitored with on‐line and off‐line metabolomics, as described in the following sections.

### 3.2 Targeted on‐line metabolomics

The three replicate *E. coli* succinate fermentation experiments were monitored with RTMet using a QQQ mass spectrometer operated in MRM mode. A total of 40 metabolites of interest were monitored, including compounds of glycolysis, the tricarboxylic acid (TCA) cycle, the pentose phosphate pathway (PPP), anaerobic fermentation, amino acids and three compounds of branched‐chained amino acid (BCAA) metabolism, previously identified as potential biomass biomarkers for this specific bioprocess (Cortada‐Garcia et al., 2022). The fermentation material automatically analyzed with RTMet was extracted via a 0.2 µm pore size biomass filtration probe and, therefore, contained extracellular medium (filtration efficiency was measured to be ≥99.98%).

**Figure 3:**
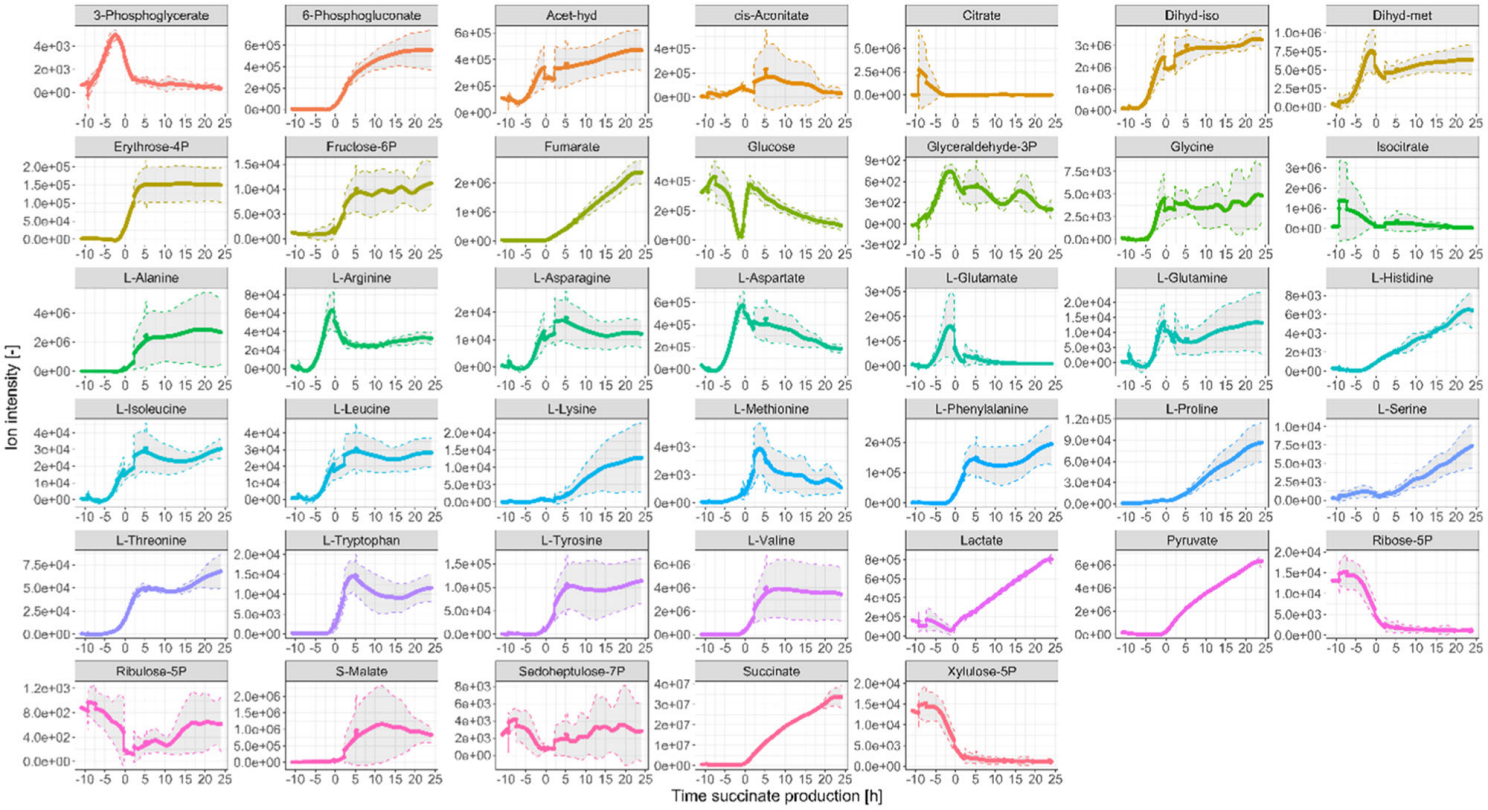
On‐line ion intensity signal for the 40 compounds monitored with RTMet. The colored lines are the average of the three replicates and the 95% confidence intervals are shown with a grey area. Time is indicated with respect to the beginning of the succinate production phase for a better comparison of the three replicates. Acet‐hyd: (S)‐2‐aceto‐2‐hydroxybutanoate; Dihyd‐iso (R)‐2,3‐dihydroxy‐isovalerate; Dihyd‐met: (R)‐2,3‐dihydroxy‐3‐methylpentanoate. Please note that due to the lack of chromatographic separation, the signal from citrate and isocitrate will partially interfere with each other despite attempting to choose MRM transitions best tailored to the individual metabolites. The same occurs for l‐glutamine and l‐lysine; and for ribose‐5P, ribulose‐5P and xylulose‐5P.

The monitoring of these metabolites is demonstrated in Figure 3, showing the evolution in time of their MS signal (ion intensity). This data is a very informative relative quantitation measurement. For example, it clearly shows how glucose is consumed until depletion during the batch phase and again during the succinate production phase once more substrate is added to the bioreactor. Moreover, succinate and other fermentative by‐products such as fumarate, lactate, pyruvate and S‐malate are mostly produced during the anaerobic phase of the bioprocess. The results also show–on one hand–an exponential increase in the abundance of many amino acids during the biomass formation phase, such as l‐arginine, l‐asparagine, l‐aspartate, l‐glutamate, l‐glutamine, l‐isoleucine and l‐leucine, which might be explained by an increased concentration of total protein in the broth caused by cellular replication. On the other hand, the production of other amino acids increases in the succinate production phase, such as l‐histidine, l‐phenylalanine, l‐proline, l‐serine, l‐threonine, l‐tryptophan and l‐tyrosine.

The three aromatic amino acids–l‐phenylalanine, l‐tryptophan and l‐tyrosine–have a common precursor, chorismate, from the shikimate pathway, which in turn requires erythrose‐4P from the PPP. Interestingly, the RTMet data also shows that the MS signals of erythrose‐4P and other sugar phosphates involved in the PPP rapidly increase soon after the beginning of the succinate production phase, such as 6‐phosphogluconate and fructose‐6P, indicating a metabolic shift in glucose consumption from being predominantly channeled down glycolysis in the aerobic batch phase, to a higher consumption via the PPP in the absence of oxygen, as it has been observed in previous LC‐MS experiments (Cortada‐Garcia et al., 2023). The metabolic reason explaining this shift could be an increased demand for reducing power during succinate production, which can be supported with NADPH formation via the oxidative PPP.

This example showcases the high level of biological and process information that can be obtained with RTMet data. The next two sections show how the RTMet MS signal can be correlated to metabolite concentration.

### 3.3 Estimation of the absolute concentration of off‐line samples

**Figure 4:**
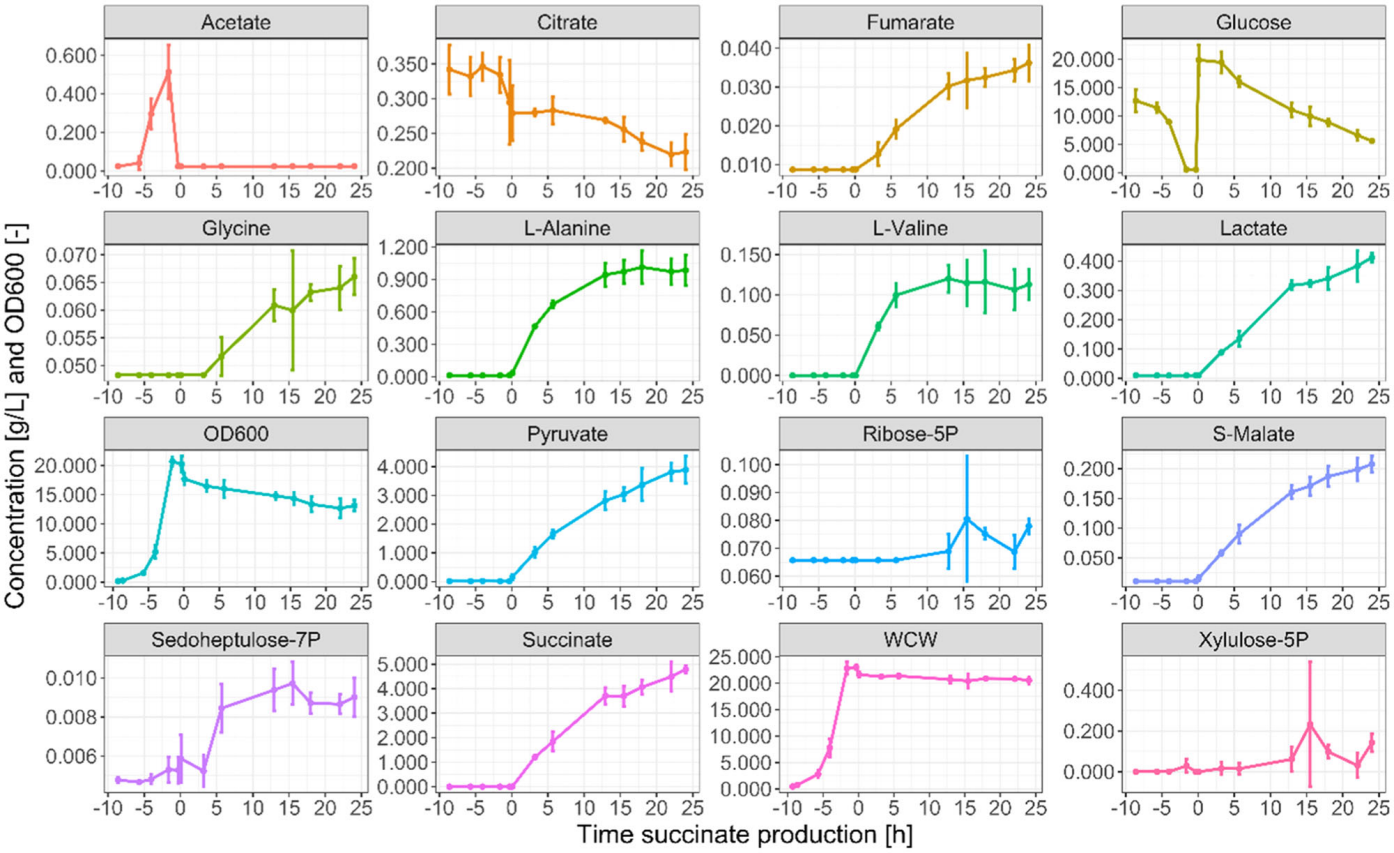
Off‐line extracellular metabolite concentrations measured by LC‐MS and off‐line biomass OD600 and wet cell weight (WCW) at the different stages of the fermentation. Time is indicated with respect to the beginning of the succinate production phase for a better comparison of the three replicates. The 95% confidence intervals are shown as error bars.

**Figure 5:**
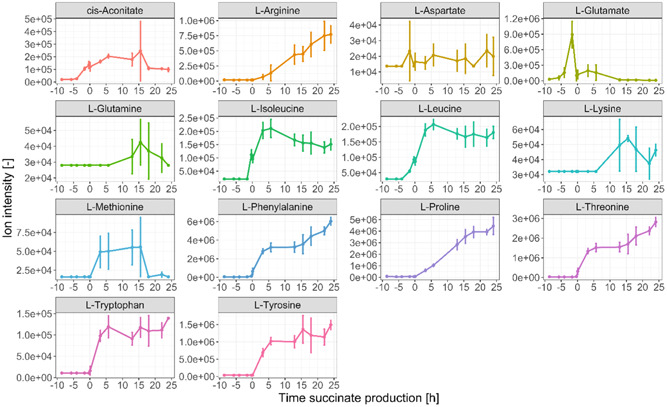
Off‐line ion intensity of the metabolites for which the concentration could not be reliably determined by LC‐MS. Time is indicated with respect to the beginning of the succinate production phase for a better comparison of the three replicates. The 95% confidence intervals are shown as error bars.

To convert the MS data monitored by RTMet into metabolite concentration, it is necessary to build a correlation model between the two types of data. While absolute quantitation of metabolites can only be determined in MS via the addition of an isotope labeled internal standard, for bioprocessing optimization it is important to incorporate absolute concentrations into process decisions. Due to the number of metabolites covered in the analysis and the impact to the instrument duty cycle caused by increasing (effectively doubling) the number of transitions, it was decided to generate standard curves using external calibration, which will provide a good estimate of the concentration with the caveat that matrix effects and ion suppression will not be taken into account. With this in mind, the concentration of metabolites of interest was measured in the extracellular medium at different time points by off‐line LC‐MS (Figure 4). Only extracellular fractions were analyzed for comparison with the RTMet measurements, which were done using a biomass filtration probe. For 14 compounds, the extracellular signal measured by LC‐MS was below their lowest point in the calibration curves, which made it not possible to reliably determine their concentration. Nevertheless, the MS signal of these 14 compounds can be followed throughout the fermentation as a relative quantitation measurement (Figure 5). Remarkably, several metabolites could be detected on‐line but not off‐line. A possible explanation could be metabolite degradation in the off‐line samples during handling, storage and freeze‐thaw cycles, which is highly minimized with RTMet.

Both Figure 4 and Figure 5 show an increase in the extracellular concentration for many metabolites during the succinate production phase. This is the case for fumarate, lactate, pyruvate, S‐malate, succinate, most amino acids and sedoheptulose‐7P. Based on their concentration, pyruvate, l‐alanine and lactate were identified as the main by‐products of succinate formation. Interestingly, 0.4 g/L of lactate are formed despite the strain carrying the genomic deletion of lactate dehydrogenase, the main gene for d‐lactate production in *E. coli* (see Figure S1 in the Supporting Information), which is probably attributable to other lactate‐producing genes in *E. coli* such as *mgsA* and *lldD* (Zhao et al., 2019).

A sudden increase in extracellular acetate and l‐glutamate is observed starting around 5 h before the beginning of the production phase, and both are consumed when the glucose is depleted. Under aerobic conditions with glucose excess, acetate is formed in *E. coli* due to overflow metabolism and the Crabtree effect, and part of this excess acetate is excreted out of the cell to prevent osmotic stress due to the accumulation of negative charges in the cytoplasm (Akesson et al., 2001; De Mey et al., 2007; Wolfe, 2005; Xu et al., 1999). l‐glutamate is one of the main ions involved in regulating osmotic pressure in gram‐negative bacteria. Roe et al. (1998) demonstrated in *E. coli* that increasing the intracellular concentration of acetate causes the cells to excrete l‐glutamate, explaining the l‐glutamate‐ peak in Figure 5.

The next section details how the concentration of metabolites measured by off‐line LC‐MS was used to develop linear regression models to correlate RTMet MS data to metabolite concentration.

### 3.4 Estimation of the absolute concentration of on‐line samples

**Figure 6:**
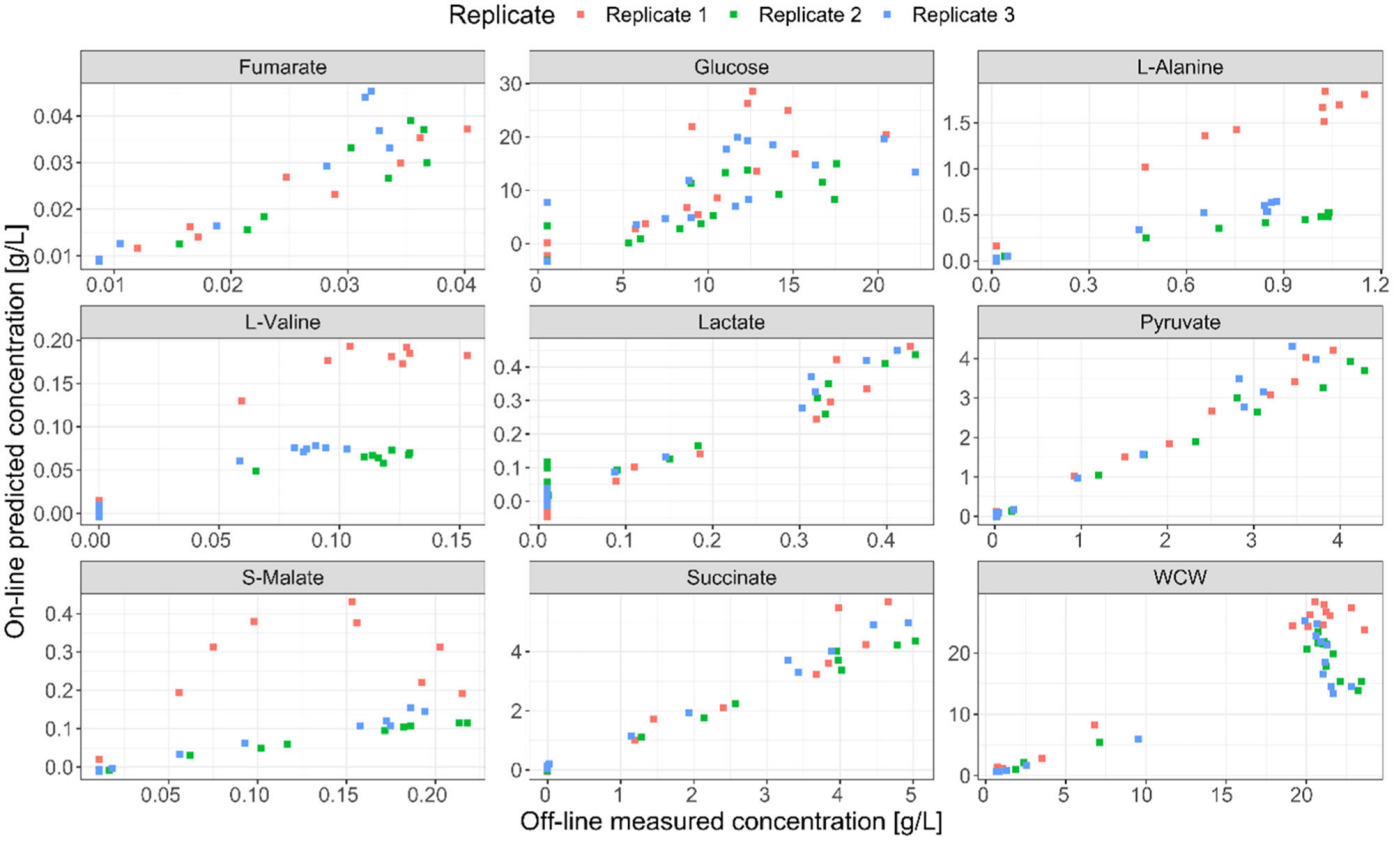
Comparison of compound concentration measured off‐line and modeled using the RTMet on‐line data. (R)2,3dihydroxyisovalerate was used as biomarker of the biomass WCW.

The three fermentation replicates were monitored with RTMet, acquiring MS data every 5 min, which was smoothed using a LOESS method. As described in the previous section, the concentration of certain metabolites of interest and the biomass were measured off‐line at different time points during the fermentation process. These 43 off‐line time points were aligned with the corresponding RTMet on‐line measurements and the two data types (on‐line MS and off‐line concentration) were correlated by linear regression using 10‐fold cross‐validation. This way, a linear model was created to correlate RTMet MS data to metabolite concentration. Furthermore, the RTMet MS data of (R)‐2,3‐dihydroxy‐isovalerate–a compound of BCAA metabolism previously identified as a biomass biomarker (Cortada‐Garcia et al., 2022)–was correlated to the off‐line WCW biomass data. This way, the on‐line monitoring of this biomarker could be used as a proxy to monitor the biomass WCW concentration. Figure 6 shows the comparison between the concentrations measured off‐line and the estimated concentrations from the RTMet data. The summary statistics of the linear regression are shown in Table 1.

**Table 1:** Parameters and summary statistics of the linear regression calibration curves correlating the off‐line concentration and the on‐line mass spectrometry signal for the eight metabolites and the biomass quantified. The on‐line (R)2,3dihydroxyisovalerate signal was used as biomarker of the off‐line biomass WCW. Metabolites not quantified because the concentration was not in range with the calibration curve are not reported.

Compound	Slope	Intercept	*R* ^2^	RMSE (g/L)	RMSRE (%)
**Fumarate**	7.5902 × 10^7^	−6.6564 × 10^5^	0.9442	0.0038	20.31
**Glucose**	1.5066 × 10^4^	6.6796 × 10^4^	0.6199	5.8890	57.34
**l‐Alanine**	2.9158 × 10^6^	−6.3663 × 10^3^	0.6750	0.3589	77.14
**l‐Valine**	3.2454 × 10^7^	1.2787 × 10^5^	0.8010	0.0360	64.05
**Lactate**	1.6151 × 10^6^	9.3153 × 10^4^	0.9646	0.0389	25.42
**Pyruvate**	1.5515 × 10^6^	4.5144 × 10^3^	0.9895	0.2537	17.07
**S‐Malate**	5.0989 × 10^6^	6.4150 × 10^4^	0.7068	0.0918	107.82
**Succinate**	6.6953 × 10^6^	4.7564 × 10^5^	0.9896	0.3682	20.73
**WCW**	1.3005 × 10^5^	‐4.7295 × 10^3^	0.8509	4.2725	26.32

Fumarate, lactate, pyruvate, and succinate show a good linear relationship between both datasets and have RMSRE values of 17%–25%. The relative error gives an overall indication of how big the RMSE of a metabolite is in comparison to its average concentration. Furthermore, these four metabolites have an *R* ^2^ higher than 0.94. Glucose, l‐alanine, l‐valine, S‐malate‐ and the biomass WCW have an *R* ^2^ 0.62–0.85. The RMSRE of glucose and WCW are between 26% and 57%, whereas for l‐alanine, l‐valine‐ and S‐malate are between 64% and 108%, which is in part caused by the low levels of these metabolites found in the extracellular fermentation medium.

Compared to similar models from the literature, Nascimento et al. (2017) developed a near‐IR (NIR) spectroscopy PLS model to monitor an ethanol fermentation and respectively reported for biomass and glucose R^2^ of cross‐validation of 0.978 and 0.920 and RMSE of cross‐validation (RMSECV) values of 0.38 and 4.65 g/L. Although their biomass predictions were more accurate, the glucose ones were similar to the ones presented here. Vann et al. (2017) also used an NIR spectroscopy PLS model to monitor an ethanol fermentation and reported an R^2^ of 0.64 for biomass, which is lower to the one obtained in this work.

Rodrigues et al. (2018) developed a mid‐IR (MIR) spectroscopy PLS model to monitor an ethanol fermentation and respectively reported for biomass and glucose an R^2^ of 0.953 and 0.998 and RMSECV values of 2.78 and 0.79 g/L, which are significantly better than the predictions from this study. Li et al. (2018) built a Raman spectroscopy PLS model to monitor a process of monoclonal antibody production using CHO cells and respectively reported for total and non‐glycosylated antibody R^2^ of cross‐validation values of 0.88 and 0.89 and RMSRE of cross‐validation (RMSRECV) values of 9% and 7% (however, these RMSREs were calculated with respect to the highest concentration of antibody instead of the average concentration, obtaining significant lower relative errors).

Overall, these examples show that similar predictive models reported in the literature commonly have *R* ^2^ values below 0.99 and similar relative errors to the ones reported here for RTMet. Although these models in the literature might at first sight seem better (higher *R* ^2^ and smaller errors), the RTMet models developed in this study have the great advantage that are simple linear models, rather than multivariate PLS models. In these multivariate models, a bigger percentage of the variance might be explained (higher *R* ^2^), but there is a higher risk of model overfitting by correlating a metabolite signal to noise or signals coming from the chemical groups of other molecules that overlap in the spectroscopic spectra. For example, Surribas et al. (2006) correlated 2D fluorescence using different excitation/emission wavelengths to glycerol production by PLS even though glycerol is not a fluorophore because glycerol production was linked to biomass formation (biomass produces a fluorescent signal). Indeed, as it has been previously mentioned, these multivariate chemometric models like PLS have limited transferability and are often only robust in the conditions they are developed. Simpler linear models like the RTMet ones developed here are expected to be more flexible and transferable to other configurations, such as different strains/organisms, bioreactors, fermentation media, temperature, and so forth.

**Figure 7:**
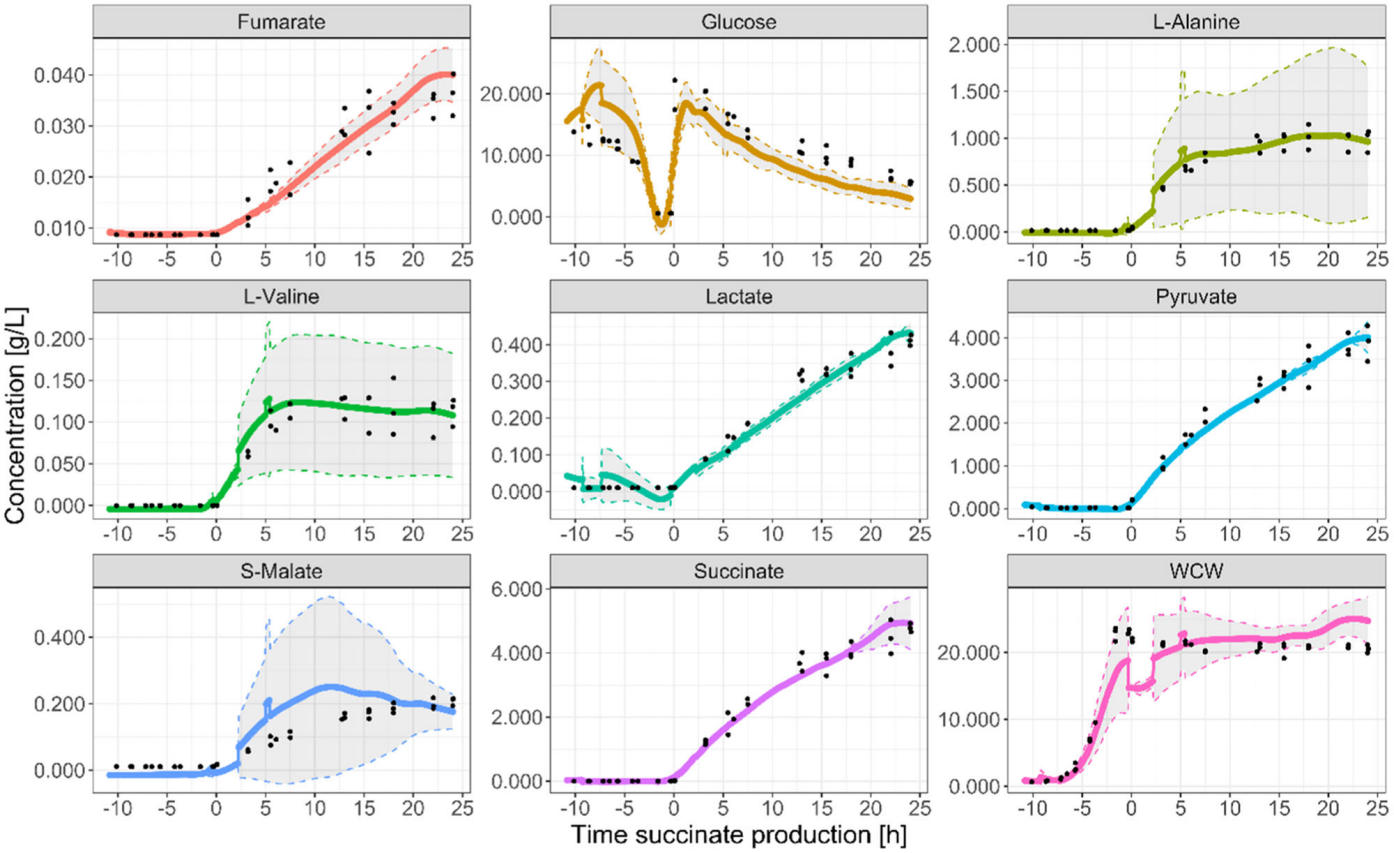
Concentration of key process metabolites and the biomass determined by RTMet (colored lines) using the calibration models for the three fermentation replicates. The colored line is the average of the three replicates and the 95% confidence intervals are shown with a grey area. The measured extracellular off‐line data for the three replicates is marked with black circles. Time is indicated with respect to the beginning of the succinate production phase for a better comparison of the three replicates.

The linear models developed were used to estimate the concentration of biomass and a few selected metabolites from the monitored RTMet data, which generally overlayed well with the off‐line measurements (Figure 7). This also indicates that the sample did not suffer any significant biological or chemical transformation during it traveling time from the bioreactor to the mass spectrometer.

Although only eight metabolites (and the biomass) are show in Figure 7, 31 additional metabolites were also monitored by RTMet in a semi‐quantitative manner, as shown in Figure 3. Indeed, the list of targeted compounds quantitatively monitored could be extended by creating additional linear regression models. Altogether, this study shows that a large number of process relevant compounds can be monitored in a quantitative or ‐semi‐quantitative way using targeted RTMet.

## 4 CONCLUSIONS

Real‐time monitoring of metabolites in a fermentation process is commonly done by spectroscopy (Raman, IR, fluorescence, etc.). The limitation of these techniques is that they commonly rely on using multivariate PLS regression models using information of several spectral peaks to successfully model metabolite concentrations. These models are labor‐ and time‐intensive to build and generally are not readily transferable to other conditions (different strains/organisms, bioreactor vessel, medium composition, process conditions, etc.). In this study, targeted on‐line metabolomics is presented as a strong alternative for metabolite monitoring, allowing the direct measurement of many compounds of interest.

Particularly, in this work 40 preselected metabolites were monitored during an *E. coli* succinate fermentation process using a QQQ mass spectrometer, including compounds of glycolysis, the TCA cycle, the PPP, metabolites of anaerobic fermentation, amino acids and biomass biomarkers. This way, metabolic regulation at different stages of the process can be identified, such as a relative increased activity in the PPP and a higher production of certain amino acids (namely l‐histidine, l‐phenylalanine, l‐proline, l‐serine, l‐threonine, l‐tryptophan and l‐tyrosine) during the anaerobic succinate production phase of the bioprocess. Importantly, the choice of metabolites monitored can be tailored to each process's needs by developing a specific MRM method using reference standards for the compounds in question.

As MS can directly measure individual compounds, RTMet allows the straight semi‐quantitative monitoring of metabolites without the need to develop correlation models. Furthermore, the absolute concentration of metabolites can be estimated using simple univariate models. In this work, the 40 target process metabolites were monitored in a semi‐quantitative way, and the absolute concentration of 8 of these–and the biomass–was estimated using linear regression. These RTMet univariate models performed comparably to vibrational spectroscopy PLS regression models reported in the literature. Although the predictions of vibrational spectroscopy generally had higher R^2^ and lower RMSE values, this is to be expected, as multivariate PLS regression will naturally explain a larger proportion of the experimental variance (by using more variables to fit the model) at the cost of a higher risk of overfitting, and hence, less robustness. Furthermore, multivariate models are also less transferable than univariate models, as they require a larger number of inputs, and dealing with missing inputs might be a challenge or compromise the model predictions.

It is important to note that adding more metabolites to the MRM method and preparing more calibration curves would allow extending the list of process compounds that can be monitored and absolute concentrations estimated using RTMet, particularly with technological advances in MS instruments that can be expected to bring ever greater analytical speed, sensitivity and gas phase separation.

## AUTHOR CONTRIBUTIONS

Joan Cortada‐Garcia and Karl Burgess conceived the idea. Joan Cortada‐Garcia did the experimental work and analyzed the data. Karl Burgess and Joan Cortada‐Garcia developed the QQQ methods. Joan Cortada‐Garcia, Jennifer Haggarty and Karl Burgess developed the RTMet system with significant assistance from Stefan Weidt. Joan Cortada‐Garcia wrote the manuscript and drew the figures. All the authors provided critical revision of the manuscript and made substantial contributions to its writing. S. Alison Arnold, Karl Burgess and Rónán Daly supervised the project and provided intellectual contributions.

## CONFLICT OF INTEREST STATEMENT

The authors declare no conflict of interest.

## Supporting information

Supporting information.

## Data Availability

The data that support the findings of this study are available in the Supporting Information and the raw data will be openly available in MetaboLights at www.ebi.ac.uk/metabolights/MTBLS7889.
