# Supplementary material for: On‐line targeted metabolomics for real‐time monitoring of relevant compounds in fermentation processes
Source: Biotechnol Bioeng. 2023 Nov 22;121(2):683–95. doi: 10.1002/bit.28599 (PMC10953439; doi:10.1002/bit.28599)
Supplement: Supplementary file 1 — Supporting information. [file BIT-121-683-s001.zip › On-line targeted metabolomics for real-time monitoring of relevant compounds in fermentation processes - Supp materials.docx]

Supporting Information

On-line targeted metabolomics for real-time monitoring of relevant compounds in fermentation processes

Joan Cortada-Garcia ^1^, Jennifer Haggarty ^2^, Stefan Weidt ^2^, Rónán Daly ^2^, S. Alison Arnold ^3^ and Karl Burgess ^1^

1 Institute of Quantitative Biology, Biochemistry and Biotechnology, School of Biological Sciences, University of Edinburgh, Edinburgh EH8 9AB, United Kingdom
2 Glasgow Polyomics, University of Glasgow, Glasgow G61 1QH, United Kingdom
3 Ingenza Ltd., Roslin Innovation Centre, Roslin EH25 9RG, United Kingdom

Table of contents

[Bacterial strain and Growth media S2](#_Toc134885461)

[Bacterial strain S2](#_Toc134885462)

[Growth media S2](#_Toc134885463)

[Succinate production metabolism S3](#_Toc134885464)

[Calibration curves for calculating the concentration of metabolites analysed by LC-MS S4](#_Toc134885465)

[Missing value imputation S6](#_Toc134885466)

[Biomass filtration probe efficiency S7](#_Toc134885467)

Bacterial strain and Growth media

Bacterial strain

All experiments described in this article were carried out using a proprietary industrial E. coli strain (Ingenza Ltd., UK), based on the E. coli NZN111 strain with deletions of the pyruvate-formate lyase (*pflB*) and lactate dehydrogenase (*ldhA*) genes as described by Chatterjee et al. (2001).

Growth media

All 5 L scale fermentation experiments were carried out with a batch phase for biomass formation using a defined minimal medium containing 11.90 g/L glucose as the main carbon source, 2.00 mM MgSO_4_, a mix of salts solution (2.00 g/L (NH_4_)_2_SO_4_, 14.60 g/L K_2_HPO_4_, 3.60 g/L NaH_2_PO_4_·2H_2_O, 0.50 g/L (NH_4_)_2_H-citrate), a mix of trace elements (1.0 mg/L CaCl_2_·2H_2_O, 20.06 mg/L FeCl_3_, 0.36 mg/L ZnSO_4_·7H_2_O, 0.32 mg/L CuSO_4_·5H_2_O, 0.30 mg/L MnSO_4_·H_2_O, 0.36 mg/L CoCl_2_·6H_2_O, 44.60 mg/L Na_2_EDTA·2H_2_O), antibiotics (100 mg/L kanamycin, 34 mg/L chloramphenicol) and antifoam (33.33 µL/L polypropylene glycol P-2000). Shake flask overnight cultures were prepared using the same medium but with 10.00 g/L glucose and no antifoam.

Succinate production metabolism

The pathway of succinate production in the industrial *E coli* strain used in this study is shown in **Figure S 1.**


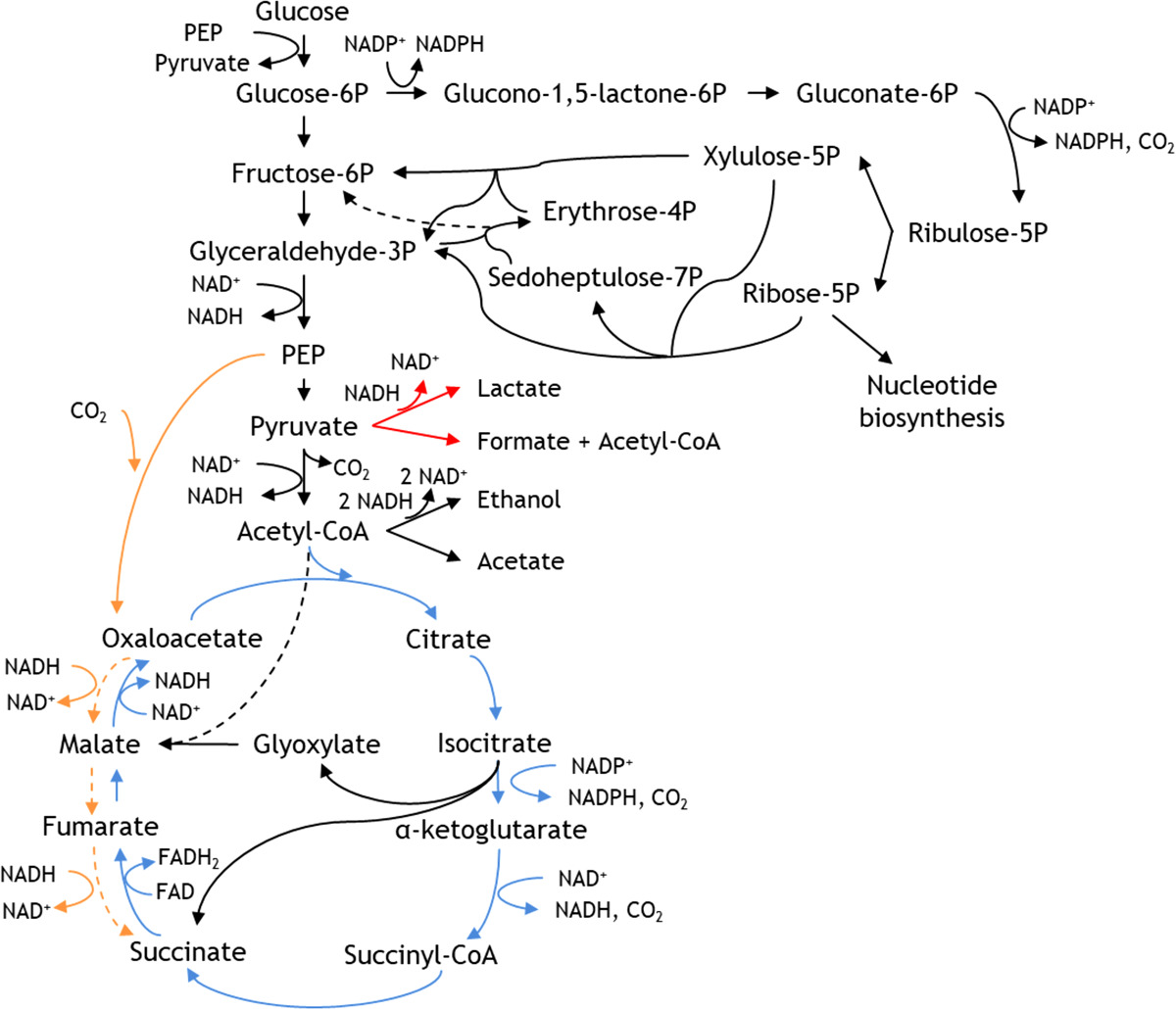


Figure S 1. Main metabolic reactions involved in succinate production in Escherichia coli. Blue lines indicate the oxidative TCA cycle under aerobic conditions. Orange lines indicate the reductive TCA cycle under anaerobic conditions. Red lines indicate deleted reactions in the industrial strain used. Arrows crossing other reactions are marked with black dashes

Calibration curves for calculating the concentration of metabolites analysed by LC-MS

A few examples of the calibration curves built to determine the metabolite concentrations by LC-MS are shown in **Figure S 2** and a summary of the calibration curve parameters for the full list of reference standards is gathered in **Table S 1**. To build these calibration curves, a series of dilutions of reference standards of the compounds of interest were analysed by LC-MS and the concentration of each compound was correlated using linear regression to the corresponding peak area measured by LC-MS.


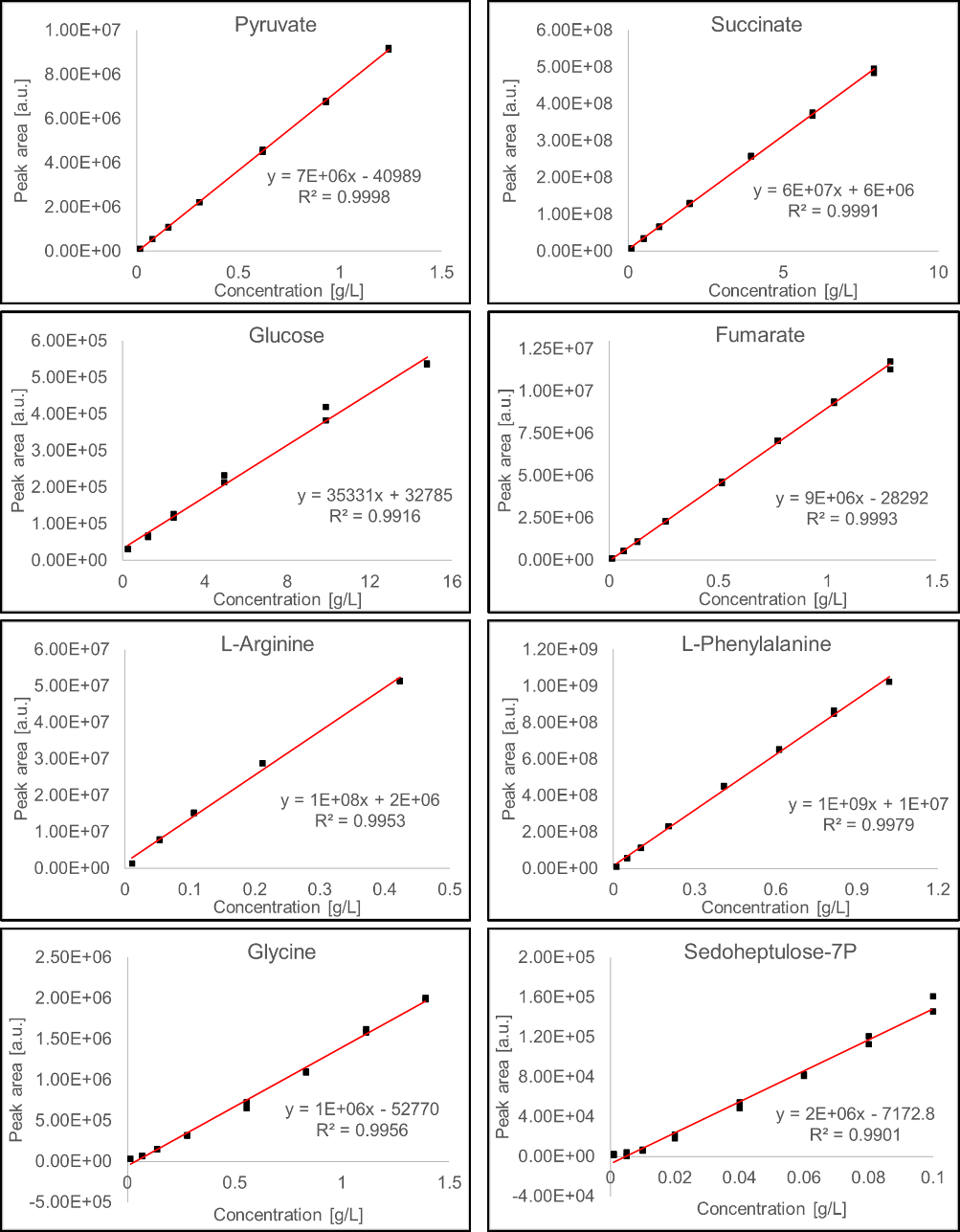


Figure S 2. Calibration curve of reference standards for determining metabolite concentration of the extracellular off-line fermentation samples. Only eight of the 28 measured metabolites are shown. The parameters for the full list of reference standards are listed in Table S 1.

Table S 1. Parameters and summary statistics of the linear regression calibration curves correlating analyte concentration and the off-line LC-MS peak area for the 28 reference standards analysed. Metabolites in the MRM method that were not found in the samples or that were not included in the list of reference standards for this analysis are not shown on the table.

| Compound | Slope | Intercept | R^2^ |
| --- | --- | --- | --- |
| Acetate | 2.05E+07 | 2.82E+06 | 0.9952 |
| cis-Aconitate | 1.84E+07 | 8.04E+05 | 0.9903 |
| Citrate | 6.51E+06 | -1.11E+06 | 0.9984 |
| DL-Lactate | 5.06E+06 | -2.97E+04 | 0.9994 |
| Fumarate | 9.05E+06 | -2.83E+04 | 0.9993 |
| Glucose | 3.53E+04 | 3.28E+04 | 0.9916 |
| Glycine | 1.45E+06 | -5.28E+04 | 0.9956 |
| L-Alanine | 2.72E+07 | -1.60E+05 | 0.9997 |
| L-Arginine | 1.20E+08 | 1.62E+06 | 0.9953 |
| L-Aspartate | 1.35E+07 | 7.91E+05 | 0.9903 |
| L-Glutamate | 1.00E+07 | 1.25E+06 | 0.9847 |
| L-Glutamine | 4.93E+08 | 6.89E+06 | 0.9919 |
| L-Isoleucine | 5.57E+07 | 4.45E+05 | 0.9986 |
| L-Leucine | 4.84E+07 | 6.12E+05 | 0.9985 |
| L-Lysine | 6.13E+08 | 6.43E+06 | 0.9923 |
| L-Methionine | 3.22E+08 | 6.74E+06 | 0.998 |
| L-Phenylalanine | 1.02E+09 | 1.49E+07 | 0.9979 |
| L-Proline | 1.02E+09 | 7.02E+07 | 0.9919 |
| L-Threonine | 4.76E+08 | 6.88E+06 | 0.9976 |
| L-Tryptophan | 2.39E+08 | 6.82E+06 | 0.9934 |
| L-Tyrosine | 1.20E+08 | 2.20E+06 | 0.992 |
| L-Valine | 2.59E+08 | 5.62E+06 | 0.9983 |
| Pyruvate | 7.39E+06 | -4.10E+04 | 0.9998 |
| Ribose-5P | 1.08E+06 | -7.08E+04 | 0.9818 |
| S-Malate | 5.97E+07 | -4.64E+05 | 0.9998 |
| Sedoheptulose-7P | 1.55E+06 | -7.17E+03 | 0.9901 |
| Succinate | 6.17E+07 | 5.63E+06 | 0.9991 |
| Xylulose-5P | 6.75E+04 | 4.10E+03 | 0.9275 |

Missing value imputation

Metabolites with missing data values were imputed using the half of the minimum intensity detected for that metabolite (Wei et al., 2018). Missing values are common in metabolomics and can happen for many different reasons, for example the metabolite being below the limit of detection or by errors occurring during data acquisition or pre-processing. In this case, it was assumed that missing values were caused by the metabolite concentration being below the limit of detection (LOD), therefore, the half-of-the-minimum intensity was used for the imputation. This method assumes that the minimum intensity detected for each metabolite is close to its LOD and that missing peaks are relatively close to (but lower than) this LOD.

Biomass filtration probe efficiency

Filtration efficiency was determined by plating 100 µL of filtered and unfiltered fermentation samples taken at the same time from the fermentation broth on selective LB agar plates containing 100 mg/L kanamycin and 34 mg/L chloramphenicol to prevent the growth of contaminants. Filtered samples were taken via the 0.2 µm filtration probe, whereas unfiltered samples were taken via the standard sampling port of the fermenter. Before plating, a series of dilutions was prepared for both samples. Filtered samples were plated neat (undiluted) and up to 10^3^x diluted. Unfiltered samples were plated 10^6^-10^8^x diluted, a much higher dilution than filtered samples, as a significantly higher concentration of cells was expected. After 24 h of incubation at 37 °C, the colony forming units (cfu) on the plates were used to calculate the filtration efficiency as shown on **Table S 2**. The filtration efficiency was measured at the two last points of the fermentation, when the biomass concentration was high and probe fouling more likely in order to obtain the worst-case estimate.

Table S 2. Measured cfu at different dilution factors for unfiltered and filtered fermentation samples used to determine the filtration efficiency of the in-line biomass filtration probe.

| Sample | Unfiltered | | | | | Filtered | | | | |  |
| --- | --- | --- | --- | --- | --- | --- | --- | --- | --- | --- | --- |
|  | **Dilution** | **Measured cfu** | **cfu x 10^dil^** | **Average cfu x 10^dil^** | **SD  cfu x 10^dil^** | **Dilution** | **Measured cfu** | **cfu x 10^dil^** | **Average cfu x 10^dil^** | **SD  cfu x 10^dil^** | **Filtration capacity [%]** |
| 1 | 6 | 232 | 2.32 x 10^8^ | 3.17 x 10^8^ | 2.70 x 10^8^ | 0 | Too many to count |  | 5.49 x 10^4^ | 1.15 x 10^4^ | 99.9827 |
| 1 | 7 | 62 | 6.20 x 10^8^ |  |  | 2 | 468 | 4.68 x 10^4^ |  |  |  |
| 1 | 8 | 1 | 1.00 x 10^8^ |  |  | 3 | 63 | 6.30 x 10^4^ |  |  |  |
| 2 | 6 | 178 | 1.78 x 10^8^ | 1.49 x 10^8^ | 4.29 x 10^7^ | 0 | Too many to count |  | 3.36 x 10^4^ | 1.61 x 10^4^ | 99.9775 |
| 2 | 7 | 17 | 1.70 x 10^8^ |  |  | 2 | 222 | 2.22 x 10^4^ |  |  |  |
| 2 | 8 | 1 | 1.00 x 10^8^ |  |  | 3 | 45 | 4.50 x 10^4^ |  |  |  |
